# Gamma heavy-chain disease accompanied with follicular lymphoma: a case report

**DOI:** 10.11613/BM.2018.010802

**Published:** 2018-02-15

**Authors:** Paula San-José, Vicente Aguadero, Granada Perea, Meritxell Estrada, Eugenio Berlanga

**Affiliations:** 1 Clinical Laboratory, Parc Taulí Hospital Universitari, Institut d’Investigació i Innovació Parc Taulí I3PT, Universitat Autònoma de Barcelona, Spain; 2 Hematology Department, Parc Taulí Hospital Universitari, Institut d’Investigació i Innovació Parc Taulí I3PT, Universitat Autònoma de Barcelona, Spain

**Keywords:** paraproteins, electrophoresis, lymphoma, heavy chain disease

## Abstract

Heavy chain diseases (HCD) are B-cell lymphoprolipherative disorders characterized by the production of monoclonal heavy chains without associated light chains. Some cases of gamma-HCD (γ-HCD) are concurrent with other lymphoid neoplasm. The monoclonal component is not always detectable by serum electrophoresis, and often an immunofixation procedure is necessary to detect this component. Prognosis is variable, and no established guidelines for follow-up are available. We describe a case of a challenging diagnosis of γ-HCD due to the absence of clinical signs frequently reported in the disease (anaemia and palatal oedema among others). Haematological malignancy was the first suspicion but bone marrow examination was negative. In addition, the presence of an autoimmune bicytopenia and a Klinefelter syndrome complicated the clinical context of the patient. A thoracoabdominal computed tomography reported many small adenopathies whose pathological and immunohystochemical study revealed a follicular lymphoma. Shortly after, serum inmunofixation secondary to an abnormal electrophoretic pattern revealed a gamma paraprotein without light chains. Eventually, γ-HCD in association with follicular lymphoma was the final diagnosis. This is the first case reporting this association.

## Introduction

Heavy chain disease (HCD) is a rare B-cell lymphoproliferative disorder characterized by the production of monoclonal, abnormally truncated, immunoglobulin heavy-chain proteins (alpha, gamma, and/or mu) without associated light chains (*1*, *2*). Heavy chain disease can be thought of as a variant of non-Hodgkin’s lymphoma. Clinical manifestations depend on the chain isotype involved and range from asymptomatic to aggressive lymphoma. Prognosis is variable, and no standardized effective treatment programs are available (*2*, *3*).

Gamma-HCD (γ-HCD), also known as Franklin’s disease, was reported for the first time in 1964. Since then, approximately 130 cases have been described in the literature (*4*). Although γ-HCD has been reported to occur equally in men and women, a slight predominance in women has recently been noted (*5*). The median age of diagnosis is 65 years old (*3*).

Classic presentation includes generalized lymphadenopathies and splenomegaly (*2*). The most distinctive symptom is palatal oedema resulting from enlargement of nodes in Waldeyer’s ring, sometimes leading to respiratory compromise (*3*, *5*).

Gamma-HCD has no specific histopathologic pattern. The most frequent histopathological finding is a pleomorphic malignant lymphoplasmacytic proliferation in bone-marrow and lymph nodes. Aspirate and biopsy specimens may show an increase in plasma cells, lymphocytes, and/or plasmacytoid lymphocytes (similar to the bone-marrow findings in Waldenström macroglobulinemia) (*3*, *6*). Nevertheless, in some cases γ-HCD are concurrent with other lymphoid neoplasms, including Hodgkin and non-Hodgkin lymphomas, which usually precede the serologic diagnosis of γ-HCD (*2*, *7*, *8*).

One third of these cases are associated with autoimmune diseases such as rheumatoid arthritis, autoimmune haemolytic anaemia, or idiopathic thrombocytopenic purpura (*5*, *6*). Some authors divide γ-HCD into three broad categories based on the underlying pathologic process and its distribution: 1) those with disseminated proliferative disease (55 - 60%); 2) those with localized proliferative disease (25%); and 3) those with no apparent proliferative disease (10 - 15%) (*3*, *6*).

In this study, we present a case of a 50-year male with gamma heavy chain disease accompanied with follicular lymphoma, a subtype of non-Hodgkin lymphoma according to WHO classification (*9*), and explain the challenge of its diagnosis.

## Case report

A 50-year-old obese, male smoker with excessive alcohol consumption and a medical history of chronic obstructive pulmonary disease was referred for a haematology consultation due to a bicytopenia detected during a routine examination in October 2016. No masses or visceromegaly were found during first physical exam. Following physical exam, haematology and biochemical analyses were performed. Samples for complete blood count (CBC) were collected from the cubital veins into blood collection Vacuette® tubes with K_3_EDTA (Greiner Bio-one, Kremsmunster, Austria) and were measured on Sysmex XN 9000 (Sysmex, Kobe, Japan) whereas peripheral blood smear was performed by microscopy. Samples for biochemical analysis were collected into blood collection Vacuette® tubes with serum clot activator and gel (Greiner Bio-one, Kremsmunster, Austria) for aspartate-aminotranspherase (AST), lactate dehydrogenase (LD), beta(β)-2 microglobulin and C-reactive protein (CRP), and were measured on the Cobas 8000 (Roche Diagnostics, Mannheim, Germany). Laboratory findings on admission are summarized in Table 1. Considering CBC, the results obtained indicated neutropenia and thrombocytopenia whereas haemoglobin was normal. A peripheral blood smear did not show dysplastic features for any haematopoietic lineage and had no circulating blasts or erythroblasts. Biochemical indicators of liver function were slightly increased, while the β-2 microglobulin concentration was above the normal values.

**Table 1:** Laboratory findings on admission and during follow up

**Test group**	**Parameter**	**Admission** **(October 2016)**	**Follow up** **(February 2017)**	**Reference intervals**
CBC	WBC (x10^9^/L)	1.3	1.33	4-11
	Neutrophils (x10^9^/L)	0.1	0.48	2.5-7.5
	Lymphocytes (x10^9^/L)	0.52	0.31	1-4.5
	Monocytes (x10^9^/L)	0.59	0.43	0.2–1.0
	Haemoglobin (g/L)	143	166	130-175
	Platelets (x10^9^/L)	54	93	130-400
Chemistry	AST (U/L)	47	44	0-38
	LD (U/L)	308	251	135-225
	β-2 MG (µg/mL)	3.7	3.9	0.8-2.2
	CRP (mg/L)	44	-	0-5
Immunology	IgG (g/L)	7.64	10.60	7.00–16.00
	IgA (g/L)	0.90	1.37	0.7–4.0
	IgM (g/L)	3.62	4.03	0.4-2.3
Serology	HBSAG	Negative	-	Negative
	HBCAC	Negative	-	Negative
	HCAC	Negative	-	Negative
	HIVAC	Negative	-	Negative
	EBIGM	Negative	-	Negative
	EBIGG	Positive	-	Negative
	CMVIGM	Negative	-	Negative
	CMVIGG	Positive	-	Negative
	MUMPSIGG	Negative	-	Negative
	MUMPSIGM	Negative	-	Negative
Autoimmunity	Anti-neutrophil antibodies	Positive*	-	Negative
	Anti-platelet antibodies	Positive^†^	-	Negative
*Presence of IgG and IgM anti-neutrophil antibodies attached to cell surface (direct test) and free in serum (indirect test). ^†^IgG anti-platelet antibodies (cut-off > 30%). CBC - complete blood count. WBC - white blood cell count. AST – aspartate-aminotransferase. LD – lactate-dehydrogenase. B-2 MG - beta-2 microglobulin. CRP - C - reactive protein. Ig – immunoglobulin. CMV – cytomegalovirus. HIV - human immunodeficiency virus. EB - Epstein-Barr virus. HBSAG - hepatitis B surface antigen index (cut-off < 1). HBCAC - anti-hepatitis B core antigen index (cut-off > 1). HCAC - hepatitis C antibody index (cut-off < 1). HIVAC - human immunodeficiency virus antibody + p24 antigen index (cut-off < 1). EBIGM - IgM antibodies to Epstein-Barr viral capsid antigens (cut-off < 20 U/mL). EBIGG - IgG antibodies to Epstein-Barr viral capsid antigens (cut-off < 20 U/mL). CMVIGM - IgM antibodies to human cytomegalovirus (cut-off < 18 U/mL). CMVIGG - IgG antibodies to human cytomegalovirus (cut-off < 12 U/mL). MUMPSIGM - IgM antibodies to mumps index (cut-off < 0.9). MUMPSIGG - IgG antibodies to mumps index (cut-off < 0.9)				

In suspicion of haematological malignancy, a bone marrow aspirate and biopsy were requested. They showed normocellular bone marrow with the presence of three haematopoietic lineages with no remarkable dysplastic features and no maturation arrest in granulocytic lineage. There was no evidence of infiltration of either tumour cells or atypical lymph cells. An incidental finding such as a chromosomal abnormality (47, XXY) was detected by cytogenetic study. He was diagnosed with Klinefelter syndrome.

Since the bone marrow study was negative, immunological examination including antinuclear antibodies, anti-mitochondrial, anti-gastric parietal cells, and anti-smooth-muscle was performed by indirect immunofluorescent tests using the commercially available kit NOVA Lite Rat (Inova Diagnostics, San Diego, California). All tests were negative. Furthermore, immunoglobulin (Ig) A, G, and M serum concentrations were investigated on Immage 800 analyser (Beckman Coulter, Brea, California). Results are presented in Table 1.

Additionally, different serological tests were examined: hepatitis and HIV on Cobas e411 (Roche diagnostics, Mannheim, Germany); mumps, Epstein-Barr virus and cytomegalovirus on Liaison (Diasorin, Saluggia, Italy). All these tests were negative (Table 1).

**Figure 1:**
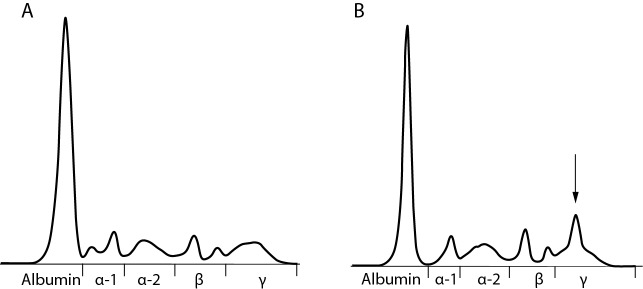
Serum electrophoresis of 50-year old male patient with heavy chain disease accompanied with follicular lymphoma on admission and after four months. A - Patient´s first serum electrophoresis showing normal pattern. B - Patient´s second serum electrophoresis after four months, with monoclonal spike in gamma globulin fraction. α-1 - alpha-1 globulin fraction. α-2 - alpha-2 globulin fraction. β - beta globulin fraction. γ - gamma globulin fraction.

Following serological examination, serum electrophoretic pattern was evaluated on Capillarys 2 (Sebia, Lisse, France). At that moment it was normal as shown in Figure 1A. Finally, presence of antibodies by immunofluorescence was investigated and results revealed presence of IgG and IgM anti-neutrophil antibodies, attached to cell surface (direct test) and free in serum (indirect test). In addition, anti-platelet antibodies analysed by flow cytometry (IgG and IgM antibodies detection by direct test) were positive (Table 1). These data introduced the possibility that the origin of bicytopenia was autoimmune.

Thoracoabdominal computed tomography (CT) performed in November 2016 showed modest splenomegaly and many small supra- and infra- diaphragmatic adenopathies, even though they were not palpable. Radiology-guided biopsy was then performed in an inguinal adenopathy. According to the pathologist, the histological sections showed a lymph node with an unstructured architecture due to the proliferation of medium to large-sized lymphocytes with vesicular chromatin and nucleoli (centroblasts) and some other smaller cells (centrocytes). The biopsy was submitted to immunohystochemical study showing cells that were positive to CD20, CD79a, BCL-6, CD10, LMO2 and BCL-2 (SP-66). It was also observed a diffuse area completely lacking follicles defined by CD21+/CD23+. It was finally defined as a follicular lymphoma grade 3 (a type of lymphoproliferative syndrome type B) based on the 2008 WHO classification.

**Figure 2:**
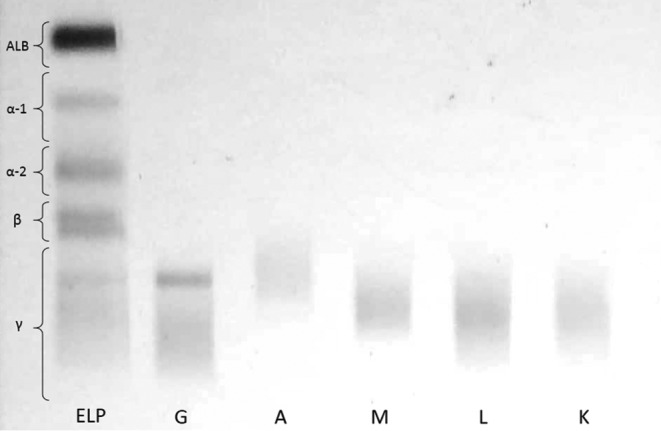
Agarose serum electrophoresis with immunofixation of the patient with gamma heavy chain disease accompanied with follicular lymphoma. Monospecific antisera were applied to five of the electrophoresis lanes as follows: G - anti-gamma heavy chain; A - anti-alpha heavy chain; M - anti-mu heavy chain; K - anti-kappa light chain; L - anti-lambda light chain. Results showing monoclonal band gamma in gamma fraction (denoted by band seen in G line) without any associated light chain monoclonal band (faint polyclonal pattern are seen in L and K lanes). ELP - electrophoretic pattern from patient. ALB - albumin region. α-1 - alpha-1 globulin region. α-2 - alpha-2 globulin region. β - beta globulin region. γ - gamma globulin region.

Four months later, in February 2017, laboratory analyses were repeated. Neutropenia and thrombocytopenia were similar (Table 1). Haemoglobin was still normal. Serum electrophoresis was performed again, and an abnormal electrophoretic pattern suggestive of a monoclonal component in the gamma region was seen (Figure 1B). Serum immunofixation was performed on Hydrasys (Sebia, Lisse, France) and revealed a gamma paraprotein with no associated monoclonal light chains (kappa or lambda) which explains the spike observed in gamma region of the electrophoresis. The serum concentration of gamma protein quantified by densitometry was 5.15 g/L (Figure 2). Electrophoresis was also performed in a 24 hour urine sample that contained 0.1 g of protein measured by Cobas 8000 (Roche Diagnostics, Mannheim, Germany) and it did not show a monoclonal spike.

Eventually, the patient was diagnosed with γ-HCD associated with lymphoproliferative syndrome type B (follicular lymphoma).

In March 2017, the patient started corticosteroid treatment (prednisone) but it stopped 10 days later due to an ascitic decompensation, probably due to an alcoholic hepatitis. At present, no haematological treatment has begun but the patient is following a close monitoring by haematologist. Once the liver problem is stabilized, the patient will be candidate for R-CHOP therapy (Rituximab with cyclophosphamide, adryamicine, vincristine and prednisone) to treat his follicular lymphoma.

## Discussion

In the present study, we describe a case of 50-year male with γ-HCD accompanied with follicular lymphoma.

Classic presentation of γ-HCD includes generalized lymphadenopathies and splenomegaly also shown by our patient by CT, but not at physical examination (*2*). One of the most distinctive symptoms is palatal oedema resulting from enlargement of nodes in Waldeyer’s ring (*4*, *5*). Our patient did not present this symptom. Although our patient did not present with anaemia, it is frequent and usually normocytic, normochromic, and moderate in γ-HCD (*3*, *5*).

Gamma-heavy chain disease has no specific histopathological pattern. The most frequent histopathological finding is a pleomorphic malignant lymphoplasmacytic proliferation in bone marrow and lymph nodes (*3*, *6*). These features of HCD infiltration were not found in our patient. Bone marrow was normal and lymph node presented a pattern of infiltration that was defined as a follicular lymphoma.

Approximately one third of described γ-HCD develop as a composite lymphoid neoplasm (*2*, *7*, *10*). In 2013, Beliauskas *et al.* made a description of 13 cases of HCD, five of which were associated to lymphoproliferative disorders (splenic diffuse red pulp small B-cell lymphoma, splenic marginal-zone lymphoma, mucosa-associated lymphoid tissue (MALT) lymphoma, lymphoplasmacytic lymphoma and monoclonal gammopathy of undetermined significance) (*2*). However, most of the cases described by these authors were associated to autoimmune disease, such as lupus erythematosus, autoimmune thyroiditis and rheumatoid arthritis (*2*). In 2016, Iijima *et al.* describe a case of γ-HCD with T-cell large granular lymphocytic leukaemia (*7*). They conducted a literature review of composite cases described since 2000. Most of them were Hodgkin lymphomas, T-cell large granular lymphocyte leukaemia, diffuse large B-cell lymphoma and splenic marginal-zone lymphoma, among others. As they remarked in their paper, most cases in the literature were classified by old criteria and lacked sufficient information about cytogenetics and histopathological pattern. It is important to emphasise that no case of follicular lymphoma has been described in association with γ-HCD in the current literature. Our case is the first reporting this association.

The patient had an autoimmune bicytopenia. Autoimmune disorders are found in about one third of γ-HCD cases, most frequently rheumatoid arthritis, but also autoimmune haemolytic anaemia, idiopathic thrombocytopenic purpura, vasculitis, Sjögren syndrome or thyroiditis (*5*, *6*, *8*). Since bone marrow examination did not show lymphoma involvement, we excluded that patient’s bicytopenia could be secondary to lymphoproliferative infiltration. Nevertheless, antibodies against neutrophils and platelets were detected suggesting the presence of an autoimmune neutropenia and thrombocytopenia. Autoimmune haemolytic anaemia and immune thrombocytopenia has been described previously in the literature associated with γ-HCD whereas immune neutropenia is not a common finding in this setting (*5*, *8*). The rest of the immunological examination, including antinuclear, anti-mitochondrial, anti-gastric parietal cells, and anti-smooth-muscle antibodies, was negative.

During the cytogenetic study, a chromosomal abnormality (47, XXY) was detected. It is common to diagnose Klinefelter syndrome in the adulthood as a consequence of infertility studies. Patients with a 47, XXY karyotype appear to have an increased risk of developing a malignancy in adulthood, usually breast cancer, extragonadal germ cell tumour and acute myeloid leukaemia. Several studies associate Klinefelter syndrome and haematological disease (*11*, *12*). Patients with excess chromosomes in cells (Down syndrome, *etc.*) may have high frequency of chromosome translocation or gene fusion during cell division (leading to the development of a haematological malignancy). Nevertheless, no evidence of association of Klinefelter syndrome and γ-HCD has been described in the literature.

Diagnosis of γ-HCD has certain difficulties and it represents an added challenge for the laboratory analyst. The monoclonal spike that appeared on the protein gel was essential for an accurate diagnosis of γ-HCD. The diagnosis can easily be missed because in 20 - 40% of cases no monoclonal spike is observed in the serum electrophoretic profile (*3*). Indeed, the first serum sample from our patient showed an electrophoretically normal pattern. Four months later, we detected a monoclonal spike that was confirmed by serum immunofixation (Figure 1). Thus, in those cases with a strong diagnostic suspicion of gammopathy or lymphoid neoplasm, we recommend serialization every 2 - 3 months of the electrophoretic serum pattern. Even if there is a normal electrophoretic profile, in these cases, we perform serum immunofixation.

The absence of free light chains can cause amount of HCD protein in the urine to be unusually small (*3*). Presently, urine samples of our patient have not presented any monoclonal component.

Upon finding a heavy chain monoclonal band in immunofixation, one should be aware that differing sensitivity of heavy and light chain reagents could potentially cause false negative results in light chain immunofixation. This would incorrectly suggest a diagnosis of HCD in cases of a regular, intact monoclonal gammopathy (*4*).

At this time, no standardized effective treatment programs for HCD are available (*2*, *3*). Most patients with low-grade lymphoplasmacytic infiltrates appear to respond to non-anthracycline-containing chemotherapy, and responses to rituximab have been reported (*9*). Some authors have described their treatment experiences. Takano *et al.* treated successfully a γ-HCD patient for the first time with rituximab in combination with standard chemotherapy (*13*). Inoue *et al.* (*14*) used a single course of combination immunochemotherapy, which consisted of rituximab and fludarabine. The patient remained in good clinical condition and transfusion independent. Our patient has not yet received any treatment until the hepatic problem is solved. Follicular lymphoma has well established treatment protocols including chemotherapy combination (anthracyclines) and anti-CD20 monoclonal therapy with rituximab.

In conclusion, γ-HCD is an extremely rare disease with a challenging diagnosis due to both uncertain clinical context and variable symptomatology. One third of cases develop as a composite lymphoid neoplasm. We have described a case of 50-year male with γ-HCD associated with follicular lymphoma for the first time. The diagnosis can easily be missed because serum protein electrophoresis does not always suggest a monoclonal gammopathy. Serum immunofixation is the only definitive procedure for demonstrating the existence of monoclonal heavy-chain paraproteins. We believe that our experience may help other professionals to handle a similar diagnosis.

## References

[1] Humeau C Monjanel H Schellenberg F Discovery of a gamma heavy chain disease in a patient followed-up for a lymphoplasma cell proliferative disorder. Ann Biol Clin (Paris). 2016;74:338–40. 27237805 10.1684/abc.2016.1149

[2] Bieliauskas S Tubbs RR Bacon CM Eshoa C Foucar K Gibson SE Gamma heavy chain disease: defining the spectrum of associated lymphoproliferative disorders through analysis of 13 cases. Am J Surg Pathol. 2012;36:534–43. 10.1097/PAS.0b013e318240590a 22301495 PMC3715127

[3] Wahner-Roedler D Kyle RA Heavy chain diseases. Best Pract Res Clin Haematol. 2005;18:729–46. 10.1016/j.beha.2005.01.029 16026747

[4] Van Keer J Meijers B Delfroge M Verhoef G Poesen K Two Cases of Heavy Chain MGUS. Case Rep Oncol Med. 2016;2016: 8749153. 10.1155/2016/8749153 27213064 PMC4861785

[5] Fermand JP Brouet JC Danon F Seligmann M Gamma Heavy Chain „Disease“: Heterogeneity of the Clinicopathologic Features. Report 16 Cases and Review of the Literature. Medicine (Baltimore). 1989;68:321–35. 10.1097/00005792-198911000-00001 2509855

[6] Wester SM Banks P Li CY The Histopathology of γ-Heavy-Chain Disease. Am J Clin Pathol. 1982;78:427–36. 10.1093/ajcp/78.4.427 6814232

[7] Iijima M Sekiguchi N Nagata A Wagatsuma M Midorikawa K Kurimoto M Gamma Heavy Chain Disease with T-cell Large Granular Lymphocytic Leukemia: A Case Report and Review of the Literature. Intern Med. 2016;55:399–403. 10.2169/internalmedicine.55.5042 26875967

[8] Wahner-Roedler DL Witzig TE Loehrer LL Kyle RA γ-Heavy-Chain Disease. Review of 23 cases. Medicine. 2003;82:236–50. 10.1097/01.md.0000085058.63483.7f 12861101

[9] Harris NL, Isaacson PG, Grogan TM, Jaffe ES. Alpha heavy chain disease. In: Swerdlow SH, Campo E, Harris NL, et al., eds. WHO classification of tumours of hematopoietic and lymphoid tissues. 4th edition. Lyon:IARC Press;2008. p.198–9.

[10] Morita K Kawamoto H Takada H Nakamura S Ishii K Okamoto Y Unusual g heavy chain disease protein in a patient with splenic marginal-zone lymphoma. Ann Clin Biochem. 2006;43:161–4. 10.1258/000456306776021490 16536920

[11] Di Benedetto G Cataldi A Verde A Gloghini A Nicolo G Pistoia V Gamma Heavy Chain Disease Associated with Hodgkin’s Disease. Clinical, Pathologic and Immunologic Features of One Case. Cancer. 1989;63:1804–9. 10.1002/1097-0142(19900501)63:9<1804::AID-CNCR2820630924>3.0.CO;2-8 2495168

[12] Keung YK Buss D Chauvenet A Pettenati M Hematologic malignancies and Klinefelter syndrome: a chance association? Cancer Genet Cytogenet. 2002;139:9–13. 10.1016/S0165-4608(02)00626-X 12547150

[13] Takano H Nagata K Mikoshiba M Nakane M Kato A Hamaguchi H Combination of rituximab and chemotherapy showing anti-tumor effect in gamma heavy chain disease expressing CD20. Letter to the editor. Am J Hematol. 2008;83:938–9. 10.1002/ajh.21284 18839436

[14] Inoue D Matsushita A Kiuchi M Takiuchi Y Nagano S Arima H Successful Treatment of g-Heavy-Chain Disease with Rituximab and Fludarabine. Acta Haematol. 2012;128:139–43. 10.1159/000339097 22890122

